# Use of in vitro and in vivo models to test candidate Alzheimer's disease risk genes from GWAS

**DOI:** 10.1002/alz70855_098683

**Published:** 2025-12-23

**Authors:** Alison M. Goate, Edoardo Marcora, Alexandra Münch, Grace Peppler, Michael Sewell, Yaima Luzardo Lightfoot, Alexandra Botte, Jim Ray

**Affiliations:** ^1^ Alzheimer Disease Research Center, New York, NY, USA; ^2^ Icahn School of Medicine at Mount Sinai, New York, NY, USA; ^3^ Ronald M. Loeb Center for Alzheimer's Disease, Icahn School of Medicine at Mount Sinai, New York, NY, USA; ^4^ The Neurodegeneration Consortium, UT MD Anderson Cancer Center, Houston, TX, USA

## Abstract

**Background:**

Genome‐wide association studies have identified many loci associated with risk for Alzheimer's disease. However, these studies rarely identify specific genes. as a result functional genomic approaches are needed to identify candidate genes and uncover mechanism underlying risk.

**Method:**

Myeloid genomics and epigenomics were used to identify candidate causal genes within AD risk loci. We then used induced pluripotent stem cell derived microglia to uncover the consequences of modulating expression of these candidate genes in vitro (microglia and triculture models (neurons/astrocytes/microglia)) and in vivo using xenotransplantation models.

**Result:**

Lower levels of MS4A4A and MS4A6A in myeloid cells are associated with lower risk for AD. RNAseq showed that MS4A4A/6A double‐knock out microglia are negatively enriched for intracellular transport and catabolic pathways and positively enriched for leukocyte immunoglobulin‐like receptor family genes. Functional studies show these cells have lower lysosomal mass compared to isogenic controls and modulation of MS4A4A/6A influences Trem2 signaling.

**Conclusion:**

MS4A4A/MS4A6A expression influences AD associated phenotypes and may be potential therpaeutic targets to lower AD risk

